# Supplementary material for: Water Influence on the Uniaxial Tensile Behavior of Polytetrafluoroethylene-Coated Glass Fiber Fabric
Source: Materials (Basel). 2021 Feb 10;14(4):846. doi: 10.3390/ma14040846 (PMC7916629; doi:10.3390/ma14040846)
Supplement: Supplementary file 1 [file materials-14-00846-s001.pdf]

materials

Supplementary Materials

Water influence on the uniaxial tensile behaviour of polytetrafluoroethylene-coated glass fibre fabric

Hastia Asadi [1,*,†], Joerg Uhlemann [1,†], Natalie Stranghoener [1,†], and Mathias Ulbricht [2,†]

[1] University of Duisburg-Essen, Institute for Metal and Lightweight Structures, Universitaetsstr. 15, 45141 Essen, Germany; joerg.uhlemann@uni-due.de (J.U.); natalie.stranghoener@uni-due.de (N.S.)
[2] University of Duisburg-Essen, Lehrstuhl für Technische Chemie II, Universitaetsstr. 7, 45117 Essen, Germany; mathias.ulbricht@uni-essen.de
* Correspondence: hastia.asadi@uni-due.de
† These authors contributed equally to this work.

Received: date; Accepted: date; Published: date

The figure numbers given in this supplementary data refer to the figure numbers of the paper and the supplementary diagrams provided here are extensions of the figures in the paper.

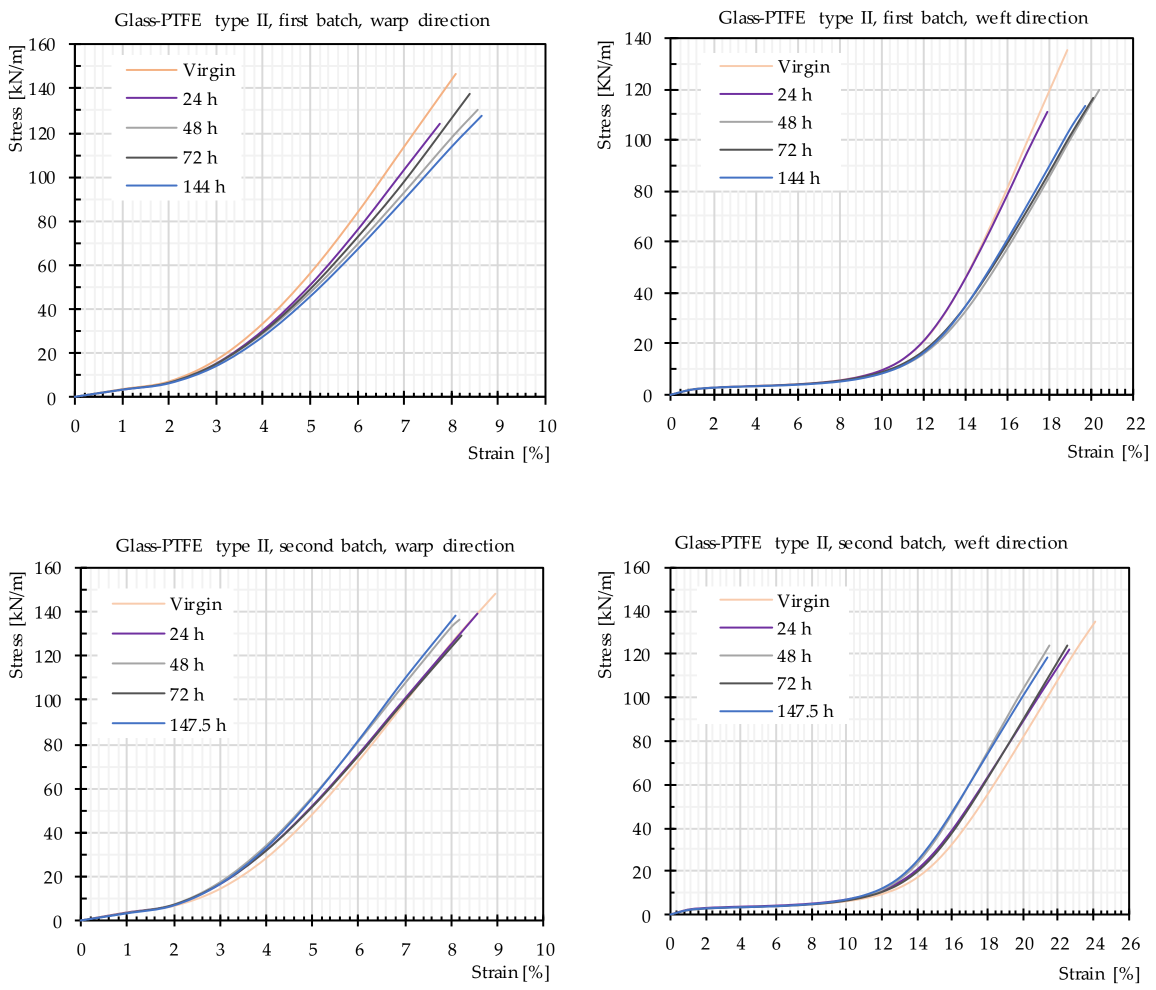

Figure 9. Stress-strain curves of glass-PTFE samples according to Table 3, in-plane and out of plane watering.

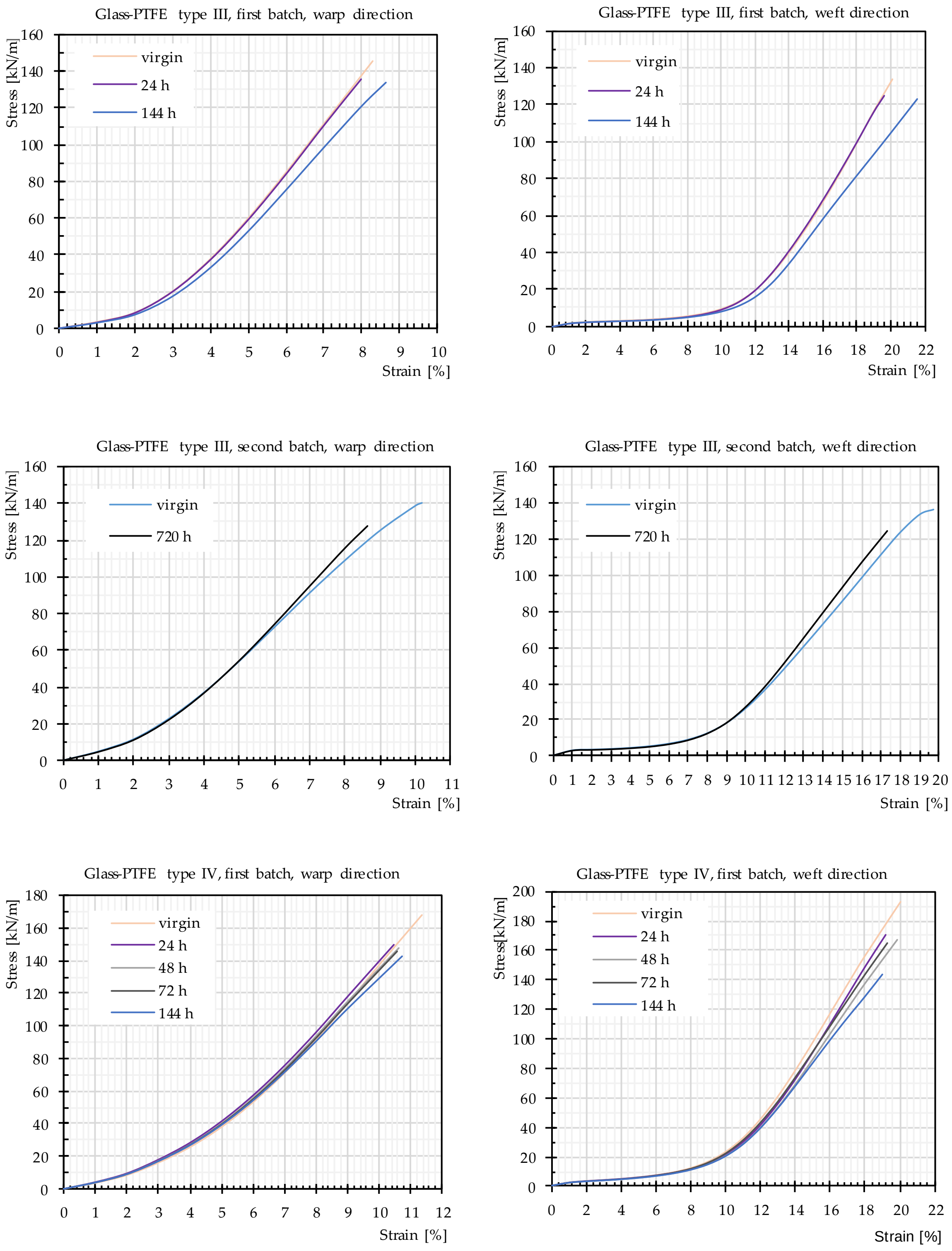

Figure 9. Stress-strain curves of glass-PTFE samples according to Table 3, in-plane and out of plane watering (Cont.).

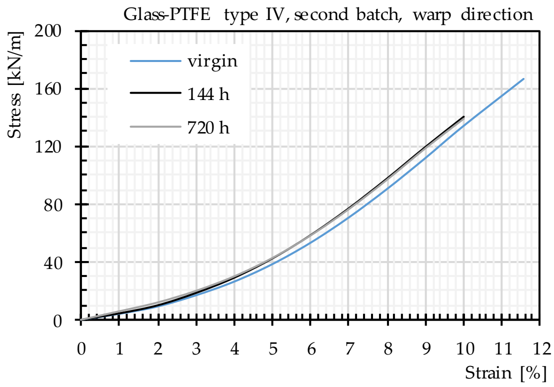

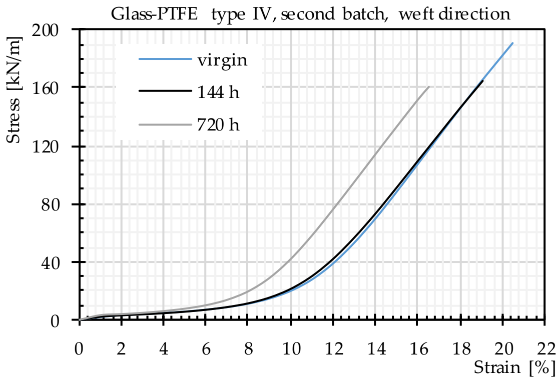

Figure 9. Stress-strain curves of glass-PTFE samples according to Table 3, in-plane and out of plane watering (Cont.).

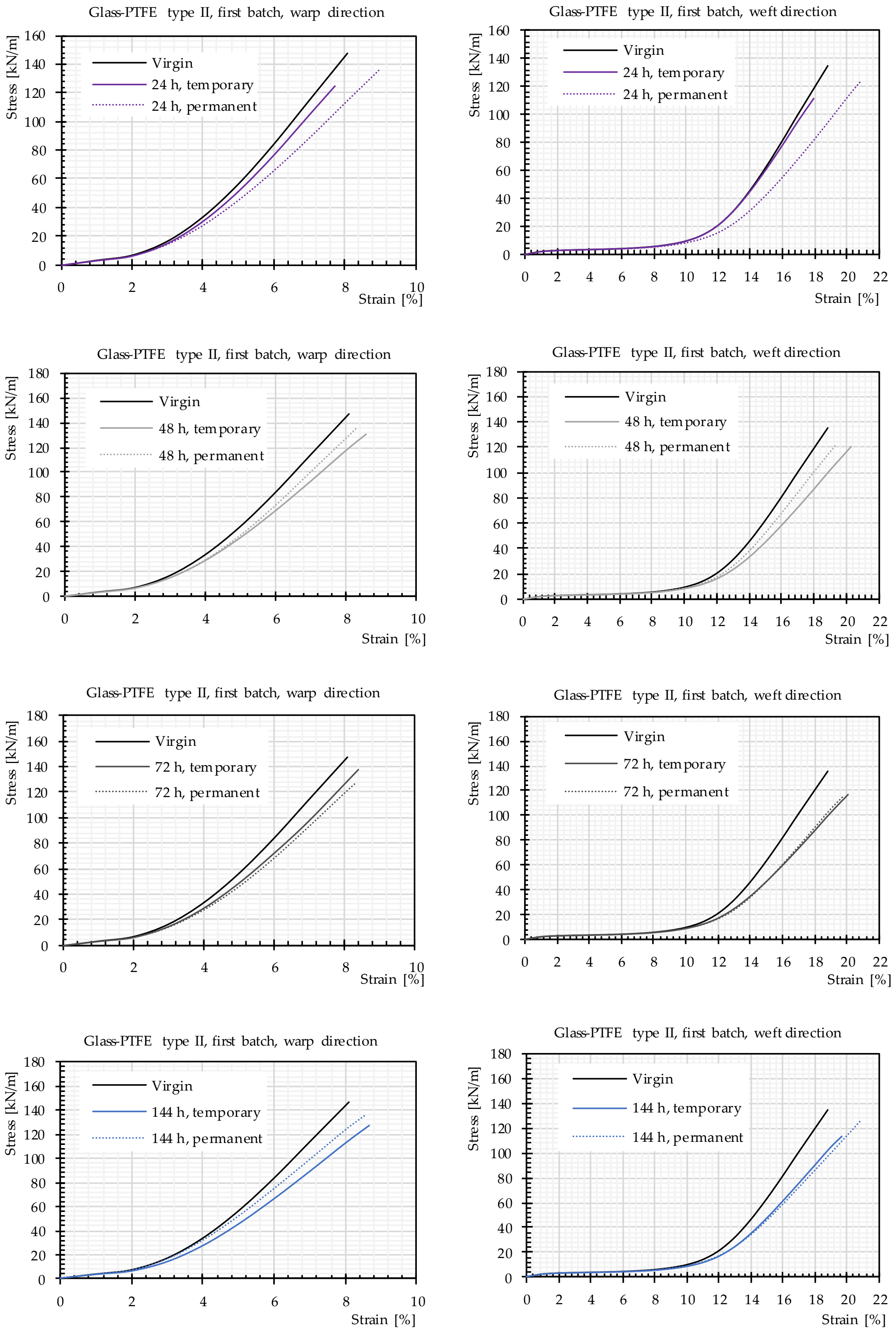

Figure 13. Permanent and temporary stress-strain curves after 24 h of watering, glass-PTFE type II first batch according to Table 3, continuous lines: temporary changes, and dashed lines: permanent changes.

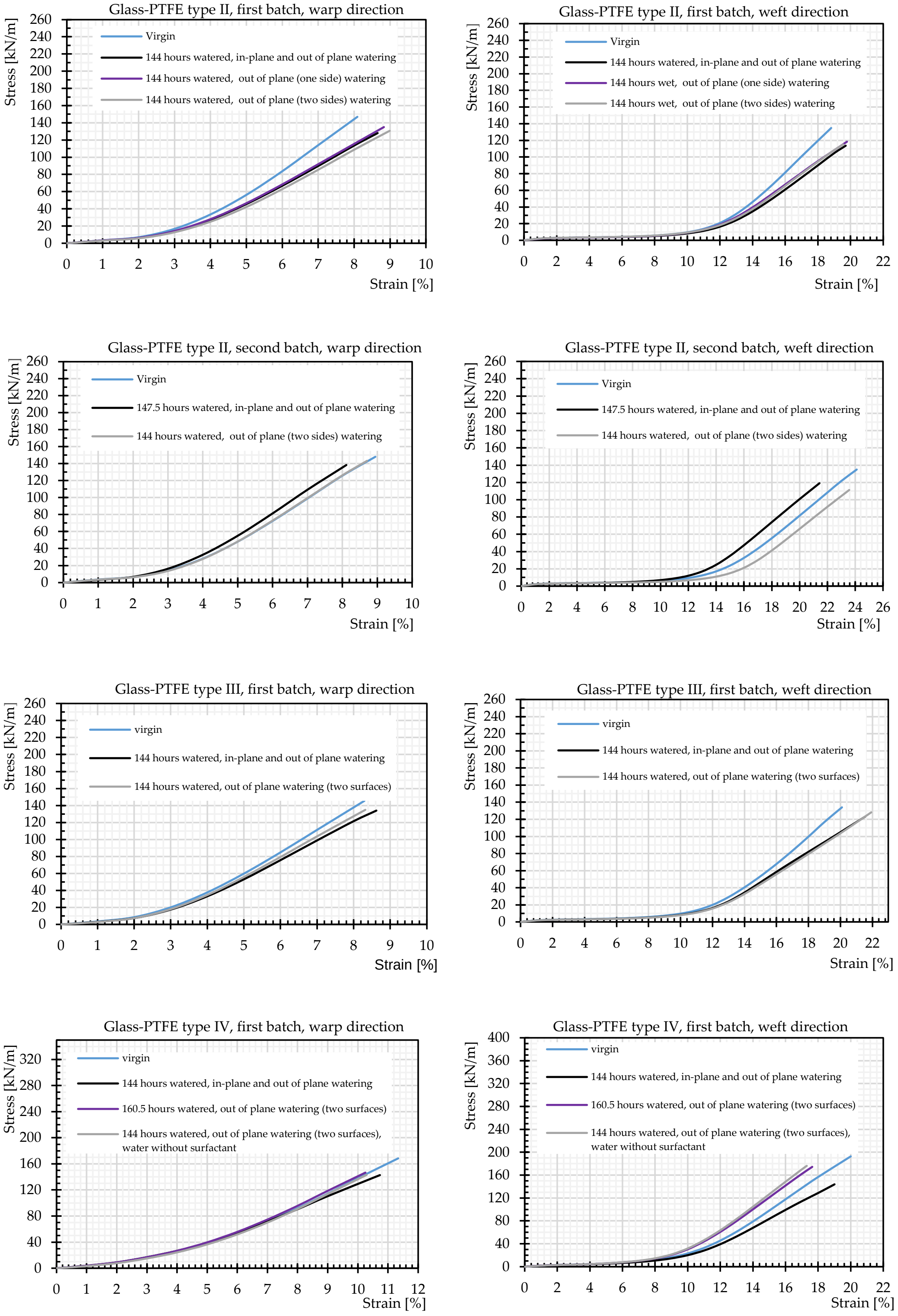

Figure 15. Stress-strain curves of strip and tank shaped specimens, glass-PTFE samples according to Table 3.

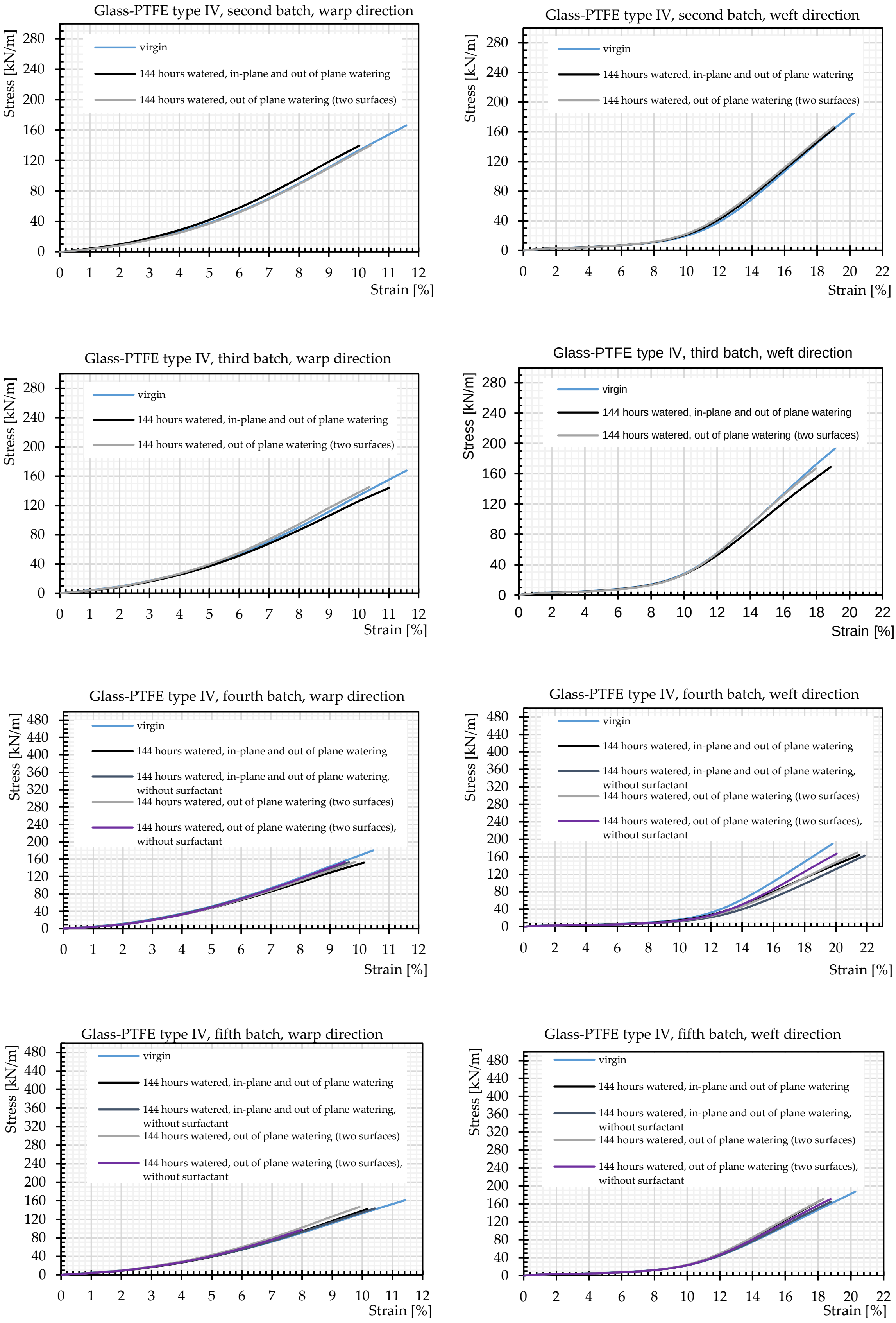

Figure 15. Stress-strain curves of strip and tank shaped specimens, glass-PTFE samples according to Table 3 (Cont.).
